# Home-Based Preoperative Exercise Training for Lung Cancer Patients Undergoing Surgery: A Feasibility Trial

**DOI:** 10.3390/jcm12082971

**Published:** 2023-04-19

**Authors:** Pedro Machado, Sara Pimenta, Ana Luís Garcia, Tiago Nogueira, Sónia Silva, Bárbara Oliveiros, Raul A. Martins, Joana Cruz

**Affiliations:** 1 Center for Innovative Care and Health Technology (ciTechCare), School of Health Sciences of the Polytechnic of Leiria (ESSLei), 2411-901 Leiria, Portugaljoana.cruz@ipleiria.pt (J.C.); 2 Univ Coimbra, Research Unit for Sport and Physical Activity (CIDAF, UID/PTD/04213/2019), Faculty of Sport Sciences and Physical Education, 3040-248 Coimbra, Portugal; raulmartins@uc.pt; 3 Physioclem, Physical Therapy Clinics, 2460-042 Alcobaça, Portugal; 4 Thoracic Surgery Unit, Portuguese Oncology Institute of Coimbra, 3000-075 Coimbra, Portugal; 5 Pulmonology Department, Leiria Hospital Center, 2410-197 Leiria, Portugal; 6 Laboratory of Biostatistics and Medical Informatics (LBIM), Faculty of Medicine, University of Coimbra, 3000-548 Coimbra, Portugal; boliveiros@fmed.uc.pt; 7 Coimbra Institute for Clinical and Biomedical Research (iCBR), Faculty of Medicine, University of Coimbra, 3000-548 Coimbra, Portugal; 8 Institute for Biomedical Imaging and Translational Research (CIBIT), University of Coimbra, 3000-548 Coimbra, Portugal

**Keywords:** prehabilitation, exercise training, lung cancer, surgical oncology, quality of life, feasibility, physical activity

## Abstract

Background: Clinical guidelines recommend prehabilitation with exercise training to optimize recovery after lung cancer surgery. However, the lack of access to facility-based exercise programs is a major barrier to routine participation. This study aimed to assess the feasibility of a home-based exercise intervention before lung cancer resection. Methods: We conducted a prospective, two-site feasibility study, including patients scheduled for lung cancer surgery. Exercise prescription involved aerobic and resistance training with telephone-based supervision. The primary endpoint was overall feasibility (recruitment rate, retention rate, intervention adherence and acceptability). Secondary endpoints included safety and effects on health-related quality of life (HRQOL) and physical performance, evaluated at baseline, after the exercise intervention and 4–5 weeks after surgery. Results: Over three months, 15 patients were eligible, and all agreed to participate (recruitment rate: 100%). A total of 14 patients completed the exercise intervention, and 12 patients were evaluated postoperatively (retention rate: 80%). The median length of the exercise intervention was 3 weeks. Patients performed an aerobic and resistance training volume higher than prescribed (median adherence rates of 104% and 111%, respectively). A total of nine adverse events occurred during the intervention (Grade 1, *n* = 8; Grade 2, *n* = 1), the most common being shoulder pain. After the exercise intervention, significant improvements were observed in the HRQOL summary score (mean difference, 2.9; 95% confidence interval [CI], from 0.9 to 4.8; *p* = 0.049) and the five-times sit-to-stand test score (median difference, −1.5; 95% CI, from −2.1 to −0.9; *p* = 0.001). After surgery, no significant effects on HRQOL and physical performance were observed. Conclusion: A short-term preoperative home-based exercise intervention is feasible before lung cancer resection and may enhance accessibility to prehabilitation. Clinical effectiveness should be investigated in future studies.

## 1. Introduction

Lung malignancy accounts for more than 11% of global cancer incidence and is the major cause of cancer-related death, with an estimate of 1.8 million deaths in 2020 [1].

Surgical resection is the therapeutic option that offers the best prognosis to lung cancer patients, with overall survival rates of 74% and 62% at 3 and 5 years after surgery, respectively [2].

However, surgery has a detrimental effect on health-related quality of life (HRQOL) [3,4], with most patients reporting limitations in their physical function and symptoms of pain, fatigue and dyspnea after lobectomy [3,5]. Furthermore, pulmonary complications such as atelectasis and pneumonia are common following lung cancer resection, increasing postoperative morbidity and mortality [6,7].

In this context, and considering that the number of lung cancer cases with an indication for surgery will increase by 60% from 2018 to 2040 [8], it is of major clinical relevance to find feasible and effective interventions that can optimize postoperative outcomes.

Prehabilitation with exercise training has been investigated in recent years, based on the rationale that it can improve patients’ physiological reserves and optimize their preparation for the stress of a tumor resection [9,10,11].

The preoperative phase represents a critical period to engage in exercise training, as the majority of early-stage lung cancer patients indicate that they would be interested in starting an exercise intervention before treatment [12]. Preoperative exercise programs have been shown to increase functional capacity and prevent pulmonary complications after lung cancer resection [13,14,15] and are recommended to enhance recovery after surgery [16].

Considering the limited time available to prepare patients for lung cancer surgery, it is critical to maximize patients’ adherence when developing preoperative exercise programs, as the effectiveness of the intervention depends on the exercise dose performed [17]. However, despite the fact that most cancer patients expressed a strong preference for exercising in a home-based environment [18,19,20] and transportation problems were the biggest barrier to participation in prehabilitation [17], research about preoperative exercise training in the context of lung cancer surgery has mainly focused on facility-based interventions, limiting patients’ accessibility [13,21,22,23,24].

Home-based exercise programs can be an alternative to meet patients’ preferences and maximize adherence to prehabilitation for lung cancer surgery, although there is still a lack of research on their feasibility [25]. To the best of the authors’ knowledge, only one study has investigated this topic [26], and it presented some limitations that weakened the translation of the findings into clinical practice. Specifically, patients were younger (mean age of 59 years) than the reported mean age for lung cancer resection (66–68 years) [2,27], not all patients had lung malignancy and there was no follow-up analysis after surgery.

The primary purpose of the present study was to determine the feasibility of a home-based exercise program (HBEP) in lung cancer patients undergoing surgical treatment. Secondary purposes were to evaluate the safety of the HBEP and explore the effects of exercise training on patients’ HRQOL and physical performance, both pre- and post-surgery.

Given that the HBEP mitigates transport barriers and addresses patients’ preferences regarding timing and setting for exercise, it was hypothesized that the intervention would be feasible before lung cancer resection.

## 2. Material and Methods

### 2.1. Design

This study was a prospective, single-arm feasibility trial conducted at the Portuguese Oncology Institute of Coimbra and Leiria Hospital Center (Portugal). Ethical approval was obtained from the Ethics Committees of the institutions involved, and the trial was registered at Clinicaltrials.gov (NCT05473052). This manuscript is reported based on the Consolidated Standards of Reporting Trials (CONSORT) guidelines, with extensions to pilot and feasibility trials [28].

### 2.2. Participants

Consecutive patients (≥18 years) were considered for inclusion if they were scheduled for surgical treatment of suspected or confirmed lung cancer (clinical stage IIIA or less), with a waiting time for surgery of at least two weeks from baseline assessment, and received medical clearance to exercise. Exclusion criteria were the following: (1) metastatic cancer; (2) presence of physical or mental disabilities that contraindicated exercise training or physical testing, evaluated by medical specialists [29,30,31] (Table S1); (3) inability to understand/speak Portuguese; (4) performing combined aerobic and resistance training over the previous month (self-reported ≥ 2 days a week, ≥30 min each session).

Eligible patients were invited to participate by their medical specialist (pulmonologist or thoracic surgeon), and those interested were introduced to the study researchers, who provided written and oral information about the trial during a scheduled outpatient appointment. All patients who agreed to participate signed a written informed consent before the initiation of any study-related procedures.

### 2.3. Intervention

**Figure 1:**
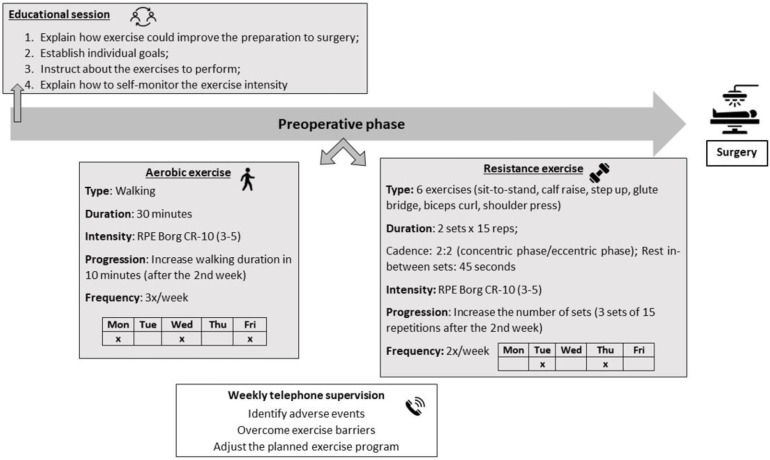
Preoperative home-based exercise program. Legend: RPE (rate of perceived exertion); Borg CR-10 (Borg Category Ratio-10); Mon (Monday); Tue (Tuesday); Wed (Wednesday); Thu (Thursday); Fri (Friday).

The HBEP involved three main components, described according to the Consensus on Exercise Reporting Template [32] (additional details provided in Table S2):

(1) Educational session: The inclusion of an educational session was based on research demonstrating that awareness of exercise benefits is a determinant for exercise behavior in cancer survivors [33]. Considering that the main motivation for lung cancer patients to participate in preoperative exercise programs is to be physically prepared for surgery [17], the goal of the educational session was to increase patients’ awareness about the potential benefits of exercise to achieve this objective. Secondly, patients were instructed by a physical therapist on how to perform the home-based exercises correctly and how to monitor exercise intensity using the Borg Category Ratio-10 (Borg CR-10) [34]. To support the explanation of each exercise, patients received a guidance document prepared by the research team that included written and photographic descriptions of the exercises [35].

(2) Aerobic and resistance exercise: The HBEP was based on international position statements about exercise in cancer care, which show that the combination of moderate-intensity aerobic and resistance training is effective to improve HRQOL [36,37]. The exercise training dose is described in Figure 1 according to the principles of Frequency, Intensity, Time and Type (F.I.T.T) [37].

Regarding the aerobic exercise component, patients were instructed to initiate a walking program of 30 min per session, 3 sessions per week in the first 2 weeks, and progress to 40 min in the following week(s). The choice of walking was based on research showing that this is the type of exercise preferred by most cancer patients [20,38,39].

The resistance exercise component was designed with the main goal of improving lower-body functional performance because this parameter is significantly associated with better HRQOL [40,41]. The resistance training incorporated 6 exercises (illustrated in Figure S1), performed in 2 sets of 15 repetitions in the first 2 weeks, progressing to 3 sets of 15 repetitions in the following week(s). The exercises were performed using bodyweight (calisthenics), a step and free-weights of one and two kilograms. Each training session started with five minutes of warm-up and ended with five minutes of stretching exercises.

To monitor training intensity, patients were instructed on how to use the Borg CR-10, which is a 10-point scale ranging from “nothing at all” to “maximal” perceived exertion [34] and is considered a valid tool to monitor and prescribe exercise intensity [42,43]. A rate of perceived exertion (RPE) between 3 and 5 (moderate to strong) was prescribed, corresponding to an intensity below or at the anaerobic threshold and approximately 40–65% of the 1-repetition maximum [34,44,45,46].

The HBEP had a flexible schedule, allowing patients who could not perform an exercise session on the planned day to compensate on another day of the week. To resemble daily clinical practice in the institutions involved, the length of the exercise program was adjusted based on the waiting times for surgery.

(3) Weekly telephone supervision: The option for this component was based on previous research showing that weekly follow-up telephone calls made by the exercise instructor were considered useful by cancer patients eligible for surgery [17]. Therefore, a physical therapist carried out weekly telephone calls with each participant to give positive reinforcement, screen for potential adverse events and recommend strategies to overcome exercise barriers that could arise during the HBEP.

### 2.4. Study Outcomes

#### 2.4.1. Primary Outcomes

Recruitment rate: Defined as the ratio of recruited patients among those who were eligible, expressed as a percentage [47].

Retention rate: Defined as the ratio of patients who completed the study among those who were recruited, expressed as a percentage [47].

Exercise adherence: Measured based on attendance rate and compliance rate. Attendance rate was defined as the ratio of the total completed-to-planned exercise sessions, expressed as a percentage [47]. Compliance rate was defined as the ratio of the total completed-to-planned training volume, expressed as a percentage.

Data regarding exercise adherence were obtained from exercise diaries, based on previous evidence indicating that diaries can be used with high validity to record exercise frequency and duration in home-based exercise interventions and have moderate-to-excellent return rates [48]. Patients were asked to record the RPE after each exercise session (Borg CR-10) and the frequency at which they completed the scheduled sessions with the planned volume (walking time and number of repetitions/sets for each resistance exercise) or to register any dose modification. Based on patients’ records in the exercise diaries, training volume was determined for each session of aerobic exercise (walking time (minutes)) and resistance exercise (sum of number of sets × number of repetitions for each exercise) [49]. Then, the session training volume was summed to quantify the total training volume. Reasons for missing sessions or exercise dose modifications were collected during weekly telephone supervisions.

Acceptability: Assessed using a 13-item questionnaire developed by the research team, based on the theoretical framework proposed by Sekhon et al. (2017) [50]. The questionnaire included questions about the different components of acceptability: (i) burden of the intervention, (ii) perceived effectiveness, (iii) intervention coherence and (iv) self-efficacy [50].

The guidelines for feasibility studies suggest that a predetermined criterion should be defined to measure the success of feasibility [51]. Therefore, based on the results of a systematic review that evaluated the feasibility of exercise interventions among lung cancer patients, a recruitment rate of 60%, a retention rate of 85% and a median adherence rate of 80% were established as study targets [47]. In terms of acceptability, the predetermined study target was an average acceptability score of 4 (scale 0–5, with 5 being the highest acceptability score).

#### 2.4.2. Secondary Outcomes

Safety: Evaluated by collecting exercise-related adverse events, defined as any unfavorable or unexpected event that occurred as a direct result of exercise training, during or within 24 h of an exercise session [47,52]. The severity of adverse events was categorized based on the Common Terminology Criteria for Adverse Events (CTCAE), version 5 [53]. The CTCAE provides a grading (severity) scale, with each adverse event being classified as Grade 1 (asymptomatic or mild symptoms, clinical or diagnostic observations only and/or intervention not indicated), Grade 2 (moderate, minimal, local or noninvasive intervention required and/or limiting age-appropriate activities of daily living), Grade 3 (severe or medically significant but not immediately life-threatening; hospitalization and/or prolongation of hospitalization indicated; disabling and limiting self-care activities of daily living), Grade 4 (life-threatening consequences and urgent intervention indicated) or Grade 5 (death) [53]. An adverse event was classified as a Serious Adverse Event (SAE) if it resulted in hospitalization, persistent or significant disability/incapacity, was life-threatening or resulted in death [47,54]. Data about adverse events were collected prospectively by one research team member (P.M.) during weekly telephone calls.

Cancer-specific health-related quality of life: Assessed through the European Organization for Research and Treatment of Cancer (EORTC) Quality of Life Questionnaire C30 (QLQ-C30) (version 3.0), a valid and reliable tool to assess HRQOL of cancer patients [55,56]. The Portuguese version of the questionnaire, which had previously been validated in 933 cancer patients with good psychometric properties [57], was used. The QLQ-C30 is composed of 30 questions, including 5 multi-item functioning scales (physical, role, cognitive, emotional and social), 3 multi-item symptom scales (fatigue, nausea and vomiting and pain), 6 single-item symptom scales (dyspnea, insomnia, appetite loss, constipation, diarrhea and financial impact), and a 2-item global health status scale (GHS) [55]. Scores range from 0 to 100 points, with higher scores in the functioning scales indicating a higher level of functioning and higher scores in the symptom scales indicating a higher level of symptom burden [55].

The EORTC QLQ-C30 summary score (SumSc) was also assessed since it provides a psychometrically more robust alternative to the GHS score that is frequently used as the primary HRQOL endpoint in clinical trials [58]. The SumSc is calculated from the mean of 13 of the 15 QLQ-C30 scores (the GHS and financial impact scales are excluded) [58].

Exercise capacity: Assessed using the Incremental Shuttle Walk Test (ISWT), which measures the distance in meters that an individual can walk around a 10 m shuttle course paced according to an incremental speed dictated by an audio recording. The test was performed under the supervision of a single assessor based on the protocol described by Singh et al. [59]. The test finished when the participant could no longer maintain the desired speed or became too breathless to continue [59]. Peripheral oxygen saturation and heart rate were monitored at rest and after test cessation using a portable pulse oximeter [60]. Patient’s dyspnea and fatigue were monitored at rest and immediately after test cessation using the Borg modified scale [60].

Handgrip strength (HGS): Assessed using a Jamar hydraulic hand dynamometer (JA Preston Corporation, Jackson, MI, USA). Measurements were conducted using the standard position approved by the American Society of Hand Therapists [61,62]. The standard adjustable handle dynamometer was set at the second handle position for all patients [61]. The non-tested arm was resting neutrally, and both feet were firmly placed on the ground, shoulder-width apart [63]. Patients were instructed to grip the handle with maximal strength for 3 s and the measurements were repeated three times for the left and right hand, with 30 s rest between measurements [61,63]. The highest value for both hands (in kilograms) was considered the output measure for each patient [64].

Five-times sit-to-stand test (5STS): Patients were instructed to perform the test on a standardized armless chair (i.e., one with a seating height between 41 and 45 cm and no elbow rests or wheels). After the instruction “ready, set, go!”, patients started the 5STS as rapidly as possible, moving from the sitting position with their buttocks touching the chair to the full standing position with their arms crossed over the chest. The 5STS finished when the patients sat on the chair after the fifth repetition, and the time needed to complete the test was registered with a stopwatch to the nearest 0.01 s [40].

### 2.5. Data Collection

Data were collected prospectively and registered in a digital database with access restrictions at three time points: T0 (baseline); T1 (after the HBEP, i.e., 2–3 days prior to surgery); and T2 (4–5 weeks after surgery). Patients were instructed to deliver the exercise diaries in the post-exercise assessment (T1), and all records were reviewed by one member of the research team (P.M.). Any doubts were discussed with the patients. The percentage of patients who returned the exercise training diaries was documented.

### 2.6. Sample Size Estimation

One aspect of feasibility studies that remains unclear is the required sample size, with recommendations suggesting that the sample size be based on key feasibility objectives [28,65]. Accordingly, as the primary aim of this study was to evaluate rates of recruitment, retention and exercise adherence, we planned to recruit 25% of the total number of lung cancer patients who are submitted annually to surgical resection in the institutions involved to ensure a desired degree of precision around the estimated rate. Given that approximately 60 patients in total are submitted annually to surgery for primary lung cancer in the institutions involved, we planned to recruit a sample size of 15 patients. This is in line with the recommendation of a sample size of 12 participants for pilot studies, based on a rationale of feasibility and precision about the mean and variance [66].

### 2.7. Statistical Analysis

Given that the objective of feasibility studies is to investigate aspects of feasibility, hypothesis testing should be secondary [67,68]. Therefore, the primary statistical analysis of this study was descriptive [67,69]. Descriptive data were expressed as mean and standard deviation (SD) or median and interquartile range (IQR) for all continuous data and counts and proportions for categorical data. Secondarily, to obtain initial estimates of the exercise training effects on HRQOL and physical performance (exercise capacity, HGS and 5STS), changes from baseline to post-exercise intervention (T1) and 4–5 weeks after surgery (T2) were analyzed using repeated measures analysis of variance (ANOVA) for normally distributed data and the Friedman’s test for non-normally distributed data. The Shapiro–Wilk test was used to check the normality of the data. When a significant time effect was found, post-hoc pairwise comparisons were conducted using Bonferroni adjustment for multiple comparisons. Additionally, the individual change in HRQOL scores from baseline to each of the subsequent timepoints was analyzed to illustrate the proportion of patients with clinically meaningful improvement or deterioration (defined for each scale as the MID) [70]. For all analyses, a *p*-value < 0.05 was considered statistically significant. All statistical analyses were performed using the SPSS package for Windows (Version 27, IBM Corporation, Chicago, IL, USA).

## 3. Results

### 3.1. Feasibility: Recruitment and Retention Rates

**Figure 2:**
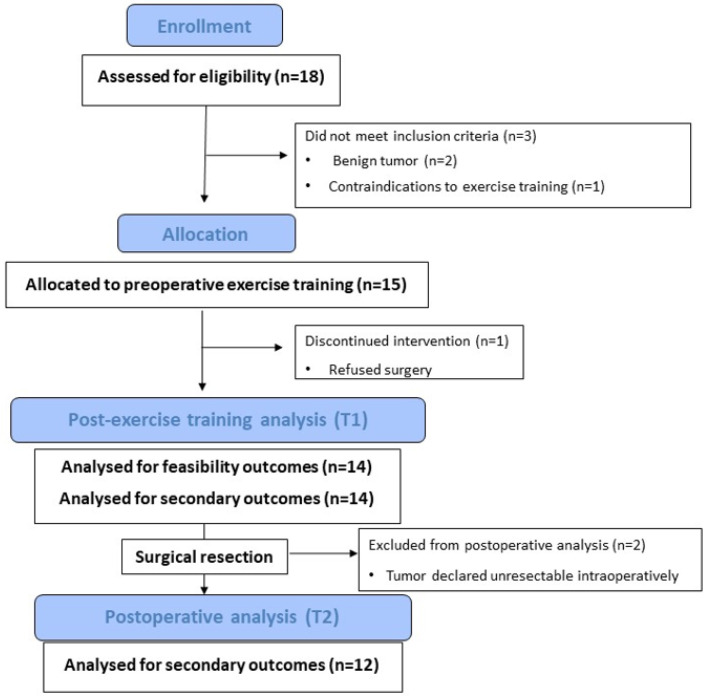
Flow diagram for the study.

The flow of patients throughout this study is summarized in Figure 2. Between February 1 and May 9, 2022, a total of 18 lung cancer patients scheduled for surgical treatment were screened for eligibility, of which 3 were not eligible. All 15 eligible patients consented to participate in the trial, which corresponds to a recruitment rate of 100% and exceeds the pre-defined study target of 60%.

The retention rate was above the pre-defined target (85%) in the preoperative period, where a total of 93% of patients (14/15 recruited) completed the HBEP and the post-exercise assessment (T1). One patient refused surgery and discontinued the intervention during the preoperative period. Two patients completed the exercise intervention and the post-exercise assessment (T1), but the tumor was declared unresectable intraoperatively and they were excluded from the postoperative analysis (T2). Hence, 80% of patients (12/15 recruited) underwent surgical resection and were included in the postoperative analysis, which was below the study target of 85% set for the retention rate.

The baseline demographic and clinical characteristics of all participants (*n* = 15) and those who underwent surgical resection (*n* = 12) are summarized in Table 1. Patients had a mean (SD) age of 67.5 (8.1) years, were predominantly male (60%) and diagnosed with adenocarcinoma (78.6%), tumor stage IA (64.3%). Among the patients submitted to surgical resection, 91.7% received a lobectomy and 8.3% a bilobectomy via video-assisted thoracoscopic surgery (50%) or an open thoracotomy (50%). The median (IQR) duration of time between the baseline assessment and surgery was 24 [23–26] days.

### 3.2. Feasibility: Exercise Adherence and Acceptability

The data regarding exercise adherence were analyzed for the 14 patients who completed the HBEP, since all of them returned the exercise diaries. Table 2 summarizes exercise adherence.

The median length of the HBEP was three weeks, ranging between two and six weeks. Overall, a total of 238 exercise sessions were completed out of 224 sessions prescribed, corresponding to a median attendance rate of 100% [IQR 93–107]. Patients completed a median of 9 sessions of aerobic exercise [IQR 8–10] and 7 sessions of resistance exercise [IQR 6–8], corresponding to a median attendance rate of 100% [IQR 89–100] and 100% [IQR 100–117], respectively.

The median compliance rate with the planned training volume was 104% for aerobic exercise [IQR 83–138] and 111% for resistance exercise [IQR 100–119]. Both the attendance rate and compliance rate exceeded the pre-defined study target of 80% set for exercise adherence.

Regarding exercise intensity, patients reported a mean (SD) RPE of 3.5 (0.2) during the sessions of aerobic exercise and a mean (SD) RPE of 3.4 (0.2) during the sessions of resistance exercise.

A total of 5 patients reported missing sessions due to bad weather (5 sessions), lack of time (2 sessions) and foot pain (1 session). Two patients required exercise dose modification before the start of the training program because of symptomatic knee osteoarthritis. One patient required the prescription of intermittent walking (3 bouts of 10 min performed throughout the day) due to preexisting hip pain that limited walking long distances. Table S3 provides a more detailed description of patients’ adherence and summarizes exercise dose modification, when required.

A total of 54 telephoned supervisions were conducted during the HBEP, corresponding to 100% of what was planned. The mean (SD) duration of each telephone supervision was 9.7 (0.9) minutes.

Regarding intervention acceptability, patients perceived the HBEP as highly acceptable, with a median average score of 4.9 [range: 4.4–5], which exceeded the pre-defined study target of an average acceptability score of 4. Globally, patients reported that the intensity of the HBEP was adjusted to their physical and mental capacity (median score of 5 [range: 3–5]) and perceived the intervention as beneficial in their preparation for surgical treatment (median score of 5 [range: 4–5]). Additionally, all patients felt confident in their ability to continue the HBEP after surgery (median score of 5 [range: 4–5]). Table S4 summarizes intervention acceptability.

### 3.3. Safety

A total of 9 adverse events were reported during the HBEP, corresponding to an incidence rate of 3.8% (9 events/238 sessions completed). No serious adverse events were reported.

The adverse events associated with resistance exercise were shoulder arthralgia (Grade 1: *n* = 4 events) and knee arthralgia (Grade 2: *n* = 1 event). Shoulder arthralgia was reported by three patients during the shoulder press exercise and was resolved after exercise modification or load reduction. Knee arthralgia was reported by one patient with pre-existing knee osteoarthritis within 24 h of the resistance exercise. This event resulted in limitations on instrumental activities of daily living and dose reduction in one session. The adverse events associated with aerobic exercise were leg muscle soreness (Grade 1: *n* = 3 events) and foot pain (Grade 1: *n* = 1 event). Leg muscle soreness was reported by two patients after the first sessions of walking and disappeared with the continuation of the HBEP. Foot pain was reported by one patient during walking and caused a missed session. No symptom exacerbation occurred in two patients with pre-existing hip pain and knee osteoarthritis. The adverse events are summarized in Table 2 and Table S3.

### 3.4. Preliminary Effects: Changes in HRQOL

**Figure 3:**
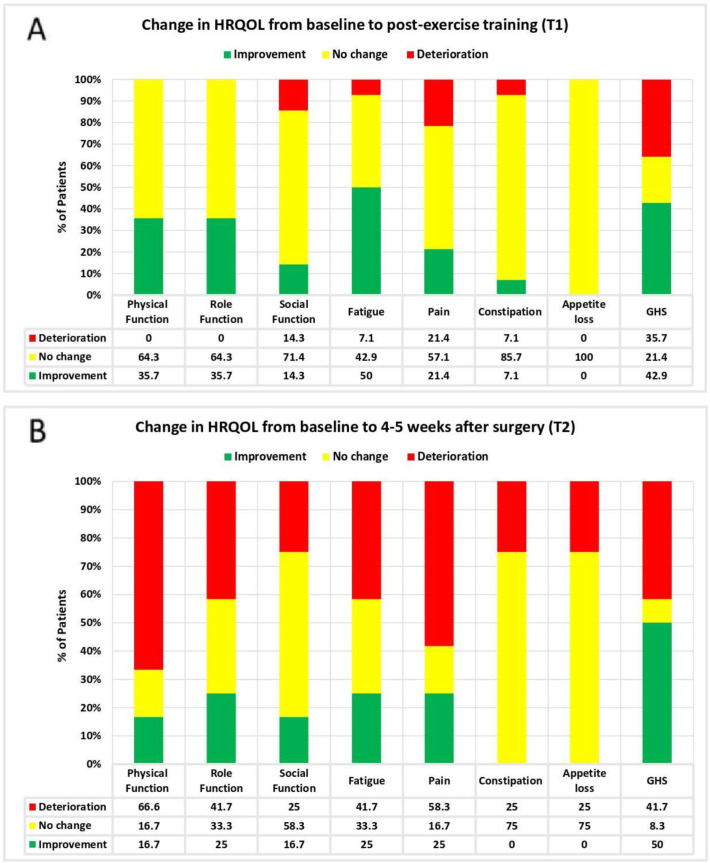
Changes in health-related quality of life based on minimal important difference. Legend: HRQOL (health-related quality of life); GHS (global health status). (**A**,**B**): Minimal important difference for improvement: global health status = 5 points; physical function = 6 points; social function = 6 points; role function = 9 points; fatigue = 6 points; pain = 9 points; appetite loss = 8 points; constipation = 13 points/Minimal important difference for deterioration: global health status = −5 points; physical function = −7 points; social function = −5 points; role function = −9 points; fatigue = −9 points; pain = −12 points; appetite loss = 8 points; constipation = −10 points [70].

Changes in HRQOL are summarized in Table 3 and Figure 3. After the HBEP (T1), there was a significant improvement in the QLQ-C30 summary score compared with the baseline (mean difference, +2.9; 95% confidence interval [CI], from 0.9 to 4.8; *p* = 0.049). Additionally, there was a trend for a significant improvement in physical functioning (mean difference, +2.8; 95% CI, from 0 to 5.6; *p* = 0.053) and fatigue (median difference, −11; 95% CI, from −16.7 to 0; *p* = 0.083). Half of the patients (50%) reported a clinically meaningful improvement in fatigue after the HBEP compared with the baseline (Figure 3A).

Postoperatively (T2), no statistically significant changes in HRQOL were observed when compared with the baseline (*p* > 0.05). However, most patients reported a clinically meaningful deterioration in physical functioning (66.6%) and pain (58.3%) (Figure 3B).

### 3.5. Preliminary Effects: Changes in Physical Performance

**Figure 4:**
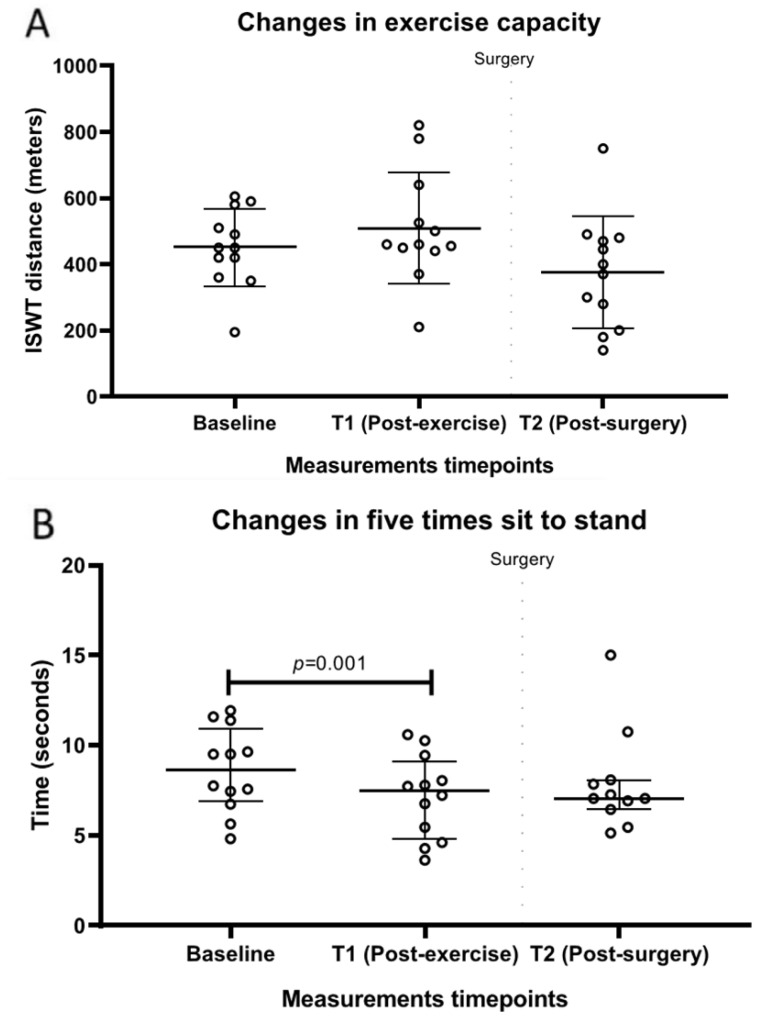
Changes in physical performance: (**A**) exercise capacity; (**B**) five-times sit-to-stand.

Changes in physical performance are summarized in Table 3 and Figure 4. After the HBEP (T1), there was a significant improvement in 5STS compared with the baseline (median difference, −1.5; 95% CI, from −2.1 to −0.9; *p* = 0.001). No other significant changes were observed either pre- or postoperatively (*p* > 0.05).

## 4. Discussion

This study evaluated the feasibility, safety and preliminary effects of a short-term HBEP in lung cancer patients undergoing surgical treatment. The main finding of the present study is that the HBEP was feasible, well accepted and safe in this clinical setting.

The rates of recruitment and exercise adherence exceeded the pre-defined study targets (60% and 80%, respectively) and were higher than the percentages reported in a previous meta-analysis for preoperative exercise interventions in lung cancer patients undergoing surgery (a median of 67% for recruitment rate and 87% for adherence rate) [47].

Of note, the median compliance rates in the current study were 104% and 111% for aerobic and resistance exercise prescriptions, respectively, meaning that the planned training volume was well tolerated by patients. Although there is a lack of studies examining the feasibility of home-based exercise training before lung cancer surgery [25], our results are similar to those observed by Coats et al. (2013) in a 4-week preoperative HBEP, reporting mean adherence rates of 125% and 83% for the aerobic and resistance exercise prescriptions, respectively [26].

The retention rate of 80% was lower than the predefined study target of 85% and inferior to the rate of 91% reported by a previous meta-analysis [47]. However, it should be noted that the retention rate after the HBEP was 93%, which is higher than the 81% reported in the study of Coats et al. (2013) [26]. Most importantly, the main reason for study discontinuation was not related to the exercise intervention but was a consequence of advanced-stage disease in two patients being declared unsuitable for surgical resection. Since the percentage of patients with an unresectable tumor was considered uncommon in the institutions involved and superior to the percentage reported in previous studies [21,23,71], this factor does not seem to represent a major obstacle to retention in a future clinical trial.

The positive results found in this study may be attributed to multiple contextual and psychosocial factors that influenced exercise adherence in cancer patients, such as: (i) matching patients’ preferences regarding timing and setting to implement exercise interventions, as early-stage lung cancer patients prefer to initiate exercise training before treatment [12] and a significant proportion of cancer patients prefer to exercise in a home-based environment [12,18,19,20], especially walking at a low-to-moderate intensity [12,20]; (ii) perceived health benefits and self-efficacy, given that all patients expressed that the intervention was beneficial in their preparation to surgical treatment and felt confident in their ability to perform the prescribed exercises. It is well established that cancer patients are more likely to adopt physical activity behaviors when they believe that the benefits associated with this behavior outweigh any perceived barriers and when they feel self-efficacious [72]; (iii) social support given during the weekly telephone calls, a component that patients found especially useful in previous prehabilitation trials [17] and a factor that is often reported as a strong facilitator to exercise engagement [72].

A total of nine non-serious adverse events were reported during the HBEP. This contrasts with the findings of Coats et al. (2013), who reported no adverse events during the exercise program [26]. An explanation for this difference may be the use of the CTCAE in the current study, which is a comprehensive tool that assesses the type and severity of adverse events in cancer patients [73]. In the study of Coats et al. (2013), the tool used to collect and grade adverse events was not reported, which possibly limited the comprehensive evaluation of the safety of the exercise intervention. Additionally, patients in the current study had a mean age of 67.5 years, compared with 59 years in the study of Coats et al. (2013) [26]. Hence, older age associated with the presence of multiple comorbidities could have contributed to a higher incidence of adverse events.

The most common adverse events in the current study were arthralgias during resistance exercise, which is consistent with a recent clinical trial reporting arthralgias in 65% of lung cancer patients performing resistance exercise [74]. However, it should be emphasized that most of the arthralgia events resolved after exercise modification or dose reduction, not compromising the completion of the HBEP. These findings highlight the need for continuous dose adjustments during the exercise program, which have also been observed in recent exercise oncology trials [74,75].

In line with previous exercise trials in lung cancer patients [47,74], no serious adverse events were observed during the HBEP, even in patients who performed an exercise dose higher than planned. This suggests that home-based exercise training is safe for lung cancer patients awaiting surgery.

Another important finding of the current study is that the QLQ-C30 summary score was significantly improved after the HBEP, suggesting exercise-induced benefits in HRQOL, mainly by a clinically relevant reduction in fatigue observed among half of the patients. This possible therapeutic effect is relevant because fatigue is highly prevalent among early-stage lung cancer patients [76,77], and poor preoperative HRQOL showed an association with prolonged hospital stays and a higher incidence of cardiopulmonary complications after lobectomy for lung cancer [78,79]. Our results are aligned with the strong evidence that exercise training improves HRQOL and fatigue among cancer patients [37,80].

In addition, a significant improvement in 5STS was found after the HBEP, indicating better lower limb functional strength. This is consistent with the findings of Coats et al. (2013), who observed an improvement in hamstring strength following home-based exercise training [26] and is of clinical importance since poor preoperative performance in the sit-to-stand test increases the risk of complications after lung cancer resection [81].

While a significant improvement was achieved in 5STS, there were no significant changes in exercise capacity (ISWT) or handgrip strength after the HBEP. These results can be partly explained by the lack of specificity of the training stimulus required to improve these outcomes, as the resistance exercise program was mainly designed to develop lower body functional strength and the walking component represented a submaximal stimulus, possibly insufficient to induce the physiological adaptations required to improve maximal exercise capacity in a restricted period of time. The study of Coats et al. (2013) supports our findings, as the authors found a significant improvement in the endurance time of a constant workrate exercise test and walking distance performed in the 6 min walk test but not in peak exercise capacity assessed by incremental cycle ergometry [26].

Most patients experienced a clinical deterioration in physical function and pain after surgery, which is in line with the results of previous observational studies [3,4,82]. However, of clinical relevance, no significant decline was observed in global health status and exercise capacity postoperatively, which contrasts with previous reports of a substantial decline in these outcomes one month after surgery [3,83,84].

While these findings suggest that the HBEP may have attenuated the deleterious effects of surgery in these clinical endpoints, as a phase I study with a small sample size and without a comparison group, it is not possible to confirm this hypothesis at this stage. Nevertheless, these exploratory results support the development of future randomized controlled trials to determine the efficacy of home-based preoperative exercise training to optimize postoperative recovery.

This study has some limitations that need to be acknowledged. Firstly, exercise adherence was assessed based on self-reported diaries, which are vulnerable to social desirability bias [85]. Nevertheless, the decision to use exercise diaries was supported by previous evidence reporting that diaries had moderate-to-excellent validity and acceptability to record adherence in home-based rehabilitation trials [48,85]. Secondly, although the acceptability questionnaire was based on the theoretical framework proposed by Sekhon et al. (2017) [50], it is not a validated tool, which may have limited a more rigorous assessment of this construct. Lastly, as a single-arm feasibility trial with a relatively small sample size, the effects of exercise training on HRQOL and physical performance should be interpreted with caution.

## 5. Conclusions

In conclusion, this trial demonstrated the feasibility of a short-term preoperative home-based exercise program before surgical treatment for lung malignancy, as evidenced by the rates of recruitment (100%), retention (80%) and intervention adherence (aerobic exercise: 104%; resistance exercise: 111%). Additionally, a safety evaluation revealed the low frequency of adverse events, all of which were resolved after exercise modification or load reduction.

Collectively, these findings suggest that the implementation of home-based exercise programs into routine clinical practice may enhance accessibility to prehabilitation for lung cancer surgery, overcoming contextual barriers associated with facility-based interventions, such as availability and transportation problems [18,19].

The role of this intervention in improving postoperative outcomes remains to be established and should be investigated in large prospective studies.

## Figures and Tables

**Table 1:** Participant demographic and clinical characteristics.

Variable	All Participants (*n* = 15)	Underwent Surgery (*n* = 12)
Age (years), mean (SD)	67.5 (8.1)	66.4 (7.2)
BMI (kg/m^2^), mean (SD)	26.3 (3.4)	26.5 (3.1)
Sex (males), no. (%)	9 (60)	7 (58.3%)
Educational level, no. (%)		
<10 years	10 (66.7)	8 (66.7)
≥10 years	5 (33.3)	3 (33.3)
Smoking status, no. (%)		
Current	7 (46.7)	5 (41.7)
Former	5 (33.3)	5 (41.7)
Never	3 (20)	2 (16.7)
Cancer diagnosis, no. (%)		
NSCLC	12 (80)	10 (83.3)
Neuroendocrine tumor	2 (13.3)	2 (16.7)
No diagnosis *	1 (6.7)	
Histological subtype ^a^, no. (%)		
Adenocarcinoma	11 (78.6)	9 (75)
Squamous cell carcinoma	1 (7.1)	1 (8.3)
Carcinoid	2 (14.3)	2 (16.7)
Tumor stage ^b^, no. (%)		
IA	9 (64.3)	8 (66.7)
IB	3 (21.4)	2 (16.7)
IIA	1 (7.1)	1 (8.3)
IIB	1 (7.1)	1 (8.3)
Comorbidities, no (%)		
Hypertension	7 (46.7)	6 (50)
Cardiovascular disease	5 (33.3)	3 (25)
COPD	4 (26.7)	4 (33.3)
Other	8 (53.3)	6 (50)
Charlson comorbidity index ^c^, mean (SD)	3.5 (1.7)	3.5 (1.6)
SpO_2_ (%), mean (SD)	96.3 (2.1)	95.8 (2.1)
Pulmonary function, mean (SD)		
FVC (% predicted)	92.1 (11.8)	93 (12.8)
FEV_1_ (% predicted)	81.9 (21.4)	81.2 (23.9)
DLCO (% predicted)	72.4 (20.8)	69.7 (22.1)
Resection degree, no. (%)		
Lobectomy		11 (91.7)
Bilobectomy		1 (8.3)
Surgical approach, no. (%)		
VATS		6 (50)
Open Surgery		6 (50)
Length of hospital stay (days), median (IQR)		3 (2.5)

Legend: BMI (body mass index); COPD (chronic obstructive pulmonary disease); DLCO (diffusion lung capacity for carbon monoxide); FEV_1_ (forced expiratory volume in one second); FVC (forced vital capacity); IQR (interquartile range); NSCLC (non-small cell lung cancer); SpO_2_ (peripheral oxygen saturation); VATS (video-assisted thoracoscopic surgery); * suspected lung cancer at baseline (patient refused surgery); ^a^ considering only patients with a confirmed diagnosis of lung cancer; ^b^ based on clinical stage at recruitment, with the exception of one patient with unstaged disease (pathological tumor stage IB). ^c^ Scores range from 0 to 24, with higher scores indicating greater comorbidities.

**Table 2:** Exercise adherence and adverse events.

Variable	Aerobic Exercise				Resistance Exercise			
	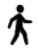				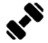			
Number of sessions, median (IQR)	9 (8–10)				7 (6–8)			
Attendance rate (%), median (IQR)	100 (89–100)				100 (100–117)			
Compliance rate (%), median (IQR)	104 (83–138)				111 (100–119)			
Weekly training volume (min/reps), median (IQR)	104 (83–145)				450 (420–500)			
Intensity (RPE) ^a^, mean (SD)	3.5 (0.2)				3.4 (0.2)			
Adverse events ^b^	Grade 1	Grade 2	Grade 3	Grade 4	Grade 1	Grade 2	Grade 3	Grade 4
Arthralgia, no. of events					4	1		
Foot pain, no. of events	1							
Leg muscle soreness, no. of events	3							
Adverse events per number of sessions completed, no. (%)	4 per 135 (3)				5 per 103 (4.9)			

Legend: IQR (interquartile range); kg (kilograms); min. (minutes); no. (number); reps (repetitions); RPE (rate of perceived exertion); SD (standard deviation); 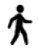 aerobic exercise; 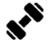 resistance exercise. ^a^ based on Borg Category Ratio scale (0–10 scale); ^b^ based on the Common Terminology Criteria for Adverse Events (Version 5.0).

**Table 3:** Measures of health-related quality of life and physical performance at each assessment timepoint.

Variable	Baseline (T0) (*n* = 12)	Post-Exercise Intervention (T1) (*n* = 12)		4–5 Weeks after Surgery (T2) (*n* = 12)		*p*-Value (Time Effect)
	Mean (SD) or Median (IQR)	Mean (SD) or Median (IQR)	Change (95% CI); ΔT1−T0	Mean (SD) or Median (IQR)	Change (95% CI); ΔT2−T0	
**HRQOL (EORTC-QLQ-C30 scales) ^§^**						
Physical Functioning ^a^	84.5 (11.4)	87.3 (10.8)	2.8 (0 to 5.6)	72.8 (16.1)	−11.6 (−26 to 2.8)	0.007 *
Role Functioning ^b^	100 (75–100)	100 (100–100)	8.3 (0 to 16.5)	91.5 (67–100)	−8.5 (−33.5 to 16.5)	0.166
Social Functioning ^b^	100 (83.3–100)	100 (100–100)	0 (−8 to 8.3)	100 (83.5–100)	0 (−25 to 8.3)	0.646
Emotional Functioning ^a^	77.1 (20.8)	79.9 (16.5)	2.9 (−8.8 to 14.6)	81.9 (15.4)	4.9 (−16.7 to 26.4)	0.662
Cognitive Functioning ^b^	100 (83.2–100)	100 (83–100)	0 (−1.7 to 8)	83 (83–100)	−0.3 (−17 to 8.3)	0.459
Global health status ^a^	62.5 (20.2)	67.4 (20.8)	4.9 (−11.4 to 21.2)	71.5 (16.5)	9 (−16.3 to 34.3)	0.498
Fatigue ^b^	22.1 (0–33)	0 (0–16.5)	−11 (−16.7 to 0)	22 (16.5 to 33)	5.4 (−11 to 27.5)	0.044 *
Pain ^b^	0 (0–8.5)	0 (0–17.5)	0 (−8 to 8.5)	17 (0–33.5)	17 (−8 to 41.5)	0.391
Dyspnea ^b^	0 (0–33.2)	0 (0–16.5)	0 (−0.2 to 0)	0 (0–33)	8.3 (−0.2 to 33)	0.129
Nausea and vomiting ^b^	0 (0)	0 (0)	0 (0 to 0)	0 (0)	0 (0 to 0)	NA
Insomnia ^b^	16.5 (0–33.2)	0 (0–33)	0 (−16.5 to 0)	16.5 (0–50)	0 (−16.7 to 33.5)	0.354
Appetite loss ^b^	0 (0–0)	0 (0–0)	0 (0 to 0)	0 (0–16.5)	0 (0 to 33.5)	NA
Constipation ^b^	0 (0–0)	0 (0–16.5)	0 (−0.2 to 0.2)	0 (0–50)	0.2 (0 to 33)	0.099
Diarrhea ^b^	0 (0)	0 (0–0)	0 (0 to 0)	0 (0–0)	0 (0 to 0)	NA
Summary Score ^a^	89.5 (6.5)	92.3 (6)	2.9 (0.9 to 4.8) *	82 (14)	−7.5 (−22 to 7)	0.049 *
**Physical performance**						
Incremental shuttle walk test (m) ^a^	451.7 (117)	511.3 (168.4)	57.5 (−5.5 to 120.5)	375.4 (170)	−76.3 (−193.3 to 40.8)	0.059
Handgrip strength, right hand (kg) ^a^	33.4 (8.3)	35.6 (9.7)	2.2 (−1 to 5.3)	34.6 (8.2)	1.2 (−2.5 to 4.8)	0.288
Handgrip strength, left hand (kg) ^a^	33.2 (10.4)	34.7 (10.3)	1.5 (0 to 3)	33.3 (9.1)	0.2 (−4.8 to 5.1)	0.434
Five-times sit-to-stand (s) ^b^	7.8 (7.1–9.6)	7.2 (5–7.9)	−1.5 (−2.1 to −0.9) *	7.1 (6.6 to 8)	−0.6 (−2.4 to 1)	0.006 *

Legend: CI (confidence interval); EORTC-QLQ-30 (European Organization for Research and Treatment of Cancer Quality of Life Questionnaire C30); HRQOL (health-related quality of life); NA (not applicable). ^§^ Higher scores in functioning scales, global health status and summary score denote better health; higher scores in symptom scales denote worse health. ^a^ Results are expressed as medians (interquartile ranges) and median change (95% CI); *p*-value was calculated using the nonparametric Friedman’s test. ^b^ Results are expressed as mean (standard deviation) and mean change (95% CI); *p*-value was calculated using the repeated measures ANOVA. * Indicates significant differences from baseline (*p* < 0.05).

## Data Availability

The data presented in this study can be obtained by contacting the corresponding author.

## Supplementary Materials

The following supporting information can be downloaded at: https://www.mdpi.com/article/10.3390/jcm12082971/s1, Figure S1: Home-based resistance exercises; Table S1: Contraindications to the preoperative exercise program; Table S2: Consensus on Exercise Reporting Template; Table S3: Exercise adherence and adverse events (individual patient data); Table S4: Acceptability of the home-based exercise program. References [29,30,31,37,50] are cited in Supplementary Materials.
