# Supplementary material for: Home-Based Preoperative Exercise Training for Lung Cancer Patients Undergoing Surgery: A Feasibility Trial
Source: J Clin Med. 2023 Apr 19;12(8):2971. doi: 10.3390/jcm12082971 (PMC10146369; doi:10.3390/jcm12082971)
Supplement: Supplementary file 1 [file jcm-12-02971-s001.zip › jcm-2272056-supplementary.pdf]

Supplementary information

Table S1. Contraindications to the preoperative exercise program

**Major signals of symptoms suggestive of cardiovascular, metabolic and renal disease**	Pain or; discomfort in the chest, neck, jaw, arms, or other areas that may result from ischemia;<br>Dizziness or syncope;<br>Orthopnea or paroxysmal nocturnal dyspnea;<br>Ankle edema;<br>Palpitations,<br>Intermittent claudication;<br>Known heart murmur;<br>Unusual fatigue or shortness of breath with usual activities
**Severe arterial hypertension**	Resting systolic blood pressure > 180 mmHg or diastolic blood pressure > .100 mmHg.
**Cardiovascular disease**	Uncontrolled symptomatic heart failure;<br>Severe ischemia of the cardiac muscle upon exertion;<br>Recent pulmonary embolism (< 3 months ago) causing severe hemodynamic strain;<br>Thrombophlebitis;<br>Acute pericarditis or myocarditis;<br>Active endocarditis;<br>Hypertrophic cardiomyopathy<br>Hemodynamically serious aortic stenosis or mitral valve stenosis;<br>Myocardial infarction less than 3 months before the start of the training program;<br>Uncontrolled cardiac dysrhythmias causing symptoms or hemodynamic compromise;<br>Suspected or known dissecting aneurysm:<br>Cerebrovascular disease resulting in transient ischaemic attacks<br>Other acute cardiovascular event less than 3 months before the start of the training program (*e.g.*, acute heart failure, acute coronary syndrome, cardiogenic shock or hypertensive crisis)
**Respiratory disease**	Pneumonia;<br>Uncontrolled asthma;
**Metabolic disease**	Uncontrolled metabolic disease (e.g., diabetes, thyrotoxicosis, or myxedema)
**Orthopedic disorders**	Lower-limb amputation without prosthesis,<br>Orthopedic disorders exacerbated by exercise training
**Other comorbidities**	Alzheimer disease;<br>Serious psychiatric disorders;<br>Severe visual or hearing disorders,<br>Uncorrected medical conditions, such as anemia or important electrolyte imbalance<br>Other mental or physical impairment leading to inability to exercise adequately and safety
**Other signals or symptoms**	Fever;<br>Dyspnea while speaking.<br>Desaturation with exercise training

Based on Fletcher et al. (2013); Riebe et al. (2015); de Rooij et al. (2014) [29–31]

Table S2 Consensus on Exercise Reporting Template

Section/Topic	Item	Checklist item	Description	Primary paper (page, table, appendix)
**WHAT: Materials**	1	Detailed description of the type of exercise equipment (e.g. weights, exercise equipment such as machines, treadmill, bicycle ergometer etc)	The exercises were performed using bodyweight (calisthenics), a step platform and free-weights of one and two kilograms.	Methods (Page 5); Illustrated Figure S1
**WHO: Provider**	2	Detailed description of the qualifications, teaching/supervising expertise, and/or training undertaken by the exercise instructor.	Physical therapist with qualifications (master degree in sport science and personal trainer certification) and expertise in exercise prescription in clinical populations.	Table S2
**HOW: Delivery**	3	Describe whether exercises are performed individually or in a group	Exercises were performed individually by each patient.	Table S2
	4	Describe whether exercises are supervised or unsupervised and how they are delivered	Sessions of walking and resistance training were performed at home (unsupervised). The exercise program included a weekly telephone supervision.	Methods (Page 5)
	5	Detailed description of how adherence to exercise is measured and reported.	Exercise adherence was measured based on attendance rate and compliance rate, using patients' records in exercise diaries.	Methods (Page 6)
	6	Detailed description of motivation strategies.	The exercise program was designed to match patients' preferences regarding exercise modality (walking) and setting (home-based program).<br>Information strategies (exercise guide and verbal explanation given during the educational session) were adopted to increase patients' awareness about the importance of regular exercise training.<br>Positive and prescriptive feedback was given during weekly telephone supervision to reinforce patients behavioral change and encourage the continuation of the training program.	Introduction (Page 3); Methods (Page 5)

	7	Detailed description of the decision rule(s) for determining exercise progression.<br><br>Detailed description of how the exercise program was progressed.	The following criteria for progression or regression in the exercise training dose were considered:<br>Criteria to regression: If patients reported a perceived exertion higher than 5 (strong) during walking or resistance training, they were advised to split the 30 minutes of walking in bouts of 10 minutes per day and/or to decrease the volume load in resistance training (reduce the number of repetitions from 15 to 10 and/or sets from two to one).<br>Criteria to progression: If patients reported a perceived exertion lower than 3 (moderate) during walking or resistance training, they were advised to increase the duration of aerobic exercise in five minutes per session and/or increase the volume load in resistance training (increase the number of repetitions from 15 to 20 and/or the number of sets from two to three).<br>If exercise dose was well tolerated during week 1 and 2 (i.e., no serious or persistent adverse events and rate of perceived exertion between 3-5 on Borg RPE CR-10 scale) patients were advised to increase the dose of walking and resistance training:<br>Walking: Increase the duration in 10 minutes (after 2nd week).<br>Resistance training: Increase the number of sets (3 sets of 15 repetitions after the 2nd week)	Methods (Page 5), Table S2
	8	Detailed description of each exercise to enable replication (e.g. photographs, illustrations , video etc)	The exercised prescribed were: sit-to-stand, step-up, calf raises, glute bridge, shoulder press and biceps curls.	Figure S1 (Illustrations and QR codes to access videos of each exercise)
	9	Detailed description of any home program component (e.g. other exercises, stretching etc)	The exercise program was entirely performed at home.	Methods (Page 5); Figure 1

	10	Describe whether there are any non-exercise components (e.g. education, cognitive behavioural therapy, massage etc)	The intervention contained an educational session, with the main goal of increase patients' awareness about the potential benefits of exercise training to improve preparation for surgery. Firstly, was emphasized that regular exercise improves health-related fitness in cancer patients (aerobic capacity and muscle strength), factors that are associated with better outcomes after surgery. Secondly, it was emphasized that regular exercise reduces fatigue in cancer patients, one of the most prevalent symptoms after surgery. To reinforce verbal explanation, patients received an exercise guide with a detailed description of the health benefits of exercise training in cancer patients.<br>During the educational session patients were also instructed on how to perform the home-based exercises correctly and how to monitor exercise intensity.	Methods (Page 4); Table S2
	11	Describe the type and number of adverse events that occurred during exercise	A total of nine non-serious adverse events were reported (Grade 1, n=8; Grade 2, n=1), predominantly arthralgias.	Results (page 10)
**WHERE: location**	12	Describe the setting in which the exercises are performed	The exercise program was performed in a home-based environment.	Methods (page 5)
**WHEN, HOW MUCH: dosage**	13	Detailed description of the exercise intervention including, but not limited to, number of exercise repetitions/sets/sessions, session duration, intervention/program duration etc.	Exercise dosage, based on F.I.T.T principle (Frequency, intensity, time and type), is detailed in Figure 1.	Methods (page 5); Figure 1
	14	14a Describe whether the exercises are generic (one size fits all) or tailored whether tailored to the individual	The exercise prescription followed the guidelines from the most recent international consensus about exercise prescription in oncology [37]. These guidelines suggest that combined moderate intensity aerobic (20-30 minutes per week; 2-3 sessions per week) and resistance training (2 sets of 8-15 reps; 2-3 sessions per week) is	Methods (Page 5); Individual adaptations are described in Results (Page 10) and Table S3

		14b Detailed description of how exercises are tailored to the individual.	effective to improve health-related quality of life in cancer patients [37] .<br>The exercise dose prescribed was tailored based on patients' musculoskeletal limitations. (Table S2).	
	15	Describe the decision rule for determining the starting level at which people commence an exercise program (such as beginner, intermediate, advanced etc)	Resistance exercise: The initial dose prescribed was 2 sets of 15 repetitions, rate of perceived rate of perceived exertion between 3–5 on the Borg RPE CR-10 scale, twice weekly. The starting external load (free-weights) was determined based on the following criteria: 1) allowing the completion of 15 repetitions with a correct technique; 2) no exercise-related pain when performing the exercise. Individual adaptations in volume load (repetitions or sets) were made to achieve a rate of perceived exertion of 3–5 on the Borg RPE CR-10 scale.<br>Aerobic exercise: The initial dose prescribed was 30 minutes of walking, rate of perceived rate of perceived exertion between 3–5 on the Borg RPE CR-10 scale, thrice weekly. Individual adaptations in walking duration (minutes) were made in patients with musculoskeletal pain exacerbated by walking or to achieve a rate of perceived exertion of 3–5 on the Borg RPE CR-10 scale.	Methods (Page 5); Individual adaptations are described in Results (Page 10) and Table S3
		16a Describe how adherence or fidelity to the exercise intervention is assessed/measured.<br>16b Describe the extent to which the intervention was delivered as planned.	Adherence to the exercise intervention was measured based on attendance rate and compliance rate, using patients' records in exercise diaries. Results are described in detail for each patient, integrating information about individual adaptations.	Results (page 9 and 10); Table 2; Table S3

Figure S1. Home-based resistance exercises

Exercise 1: *Step up*

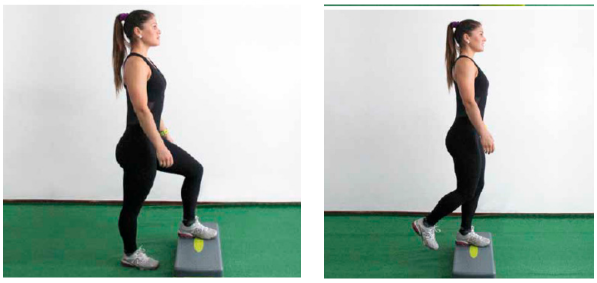

Exercise 2: *Glute bridge*

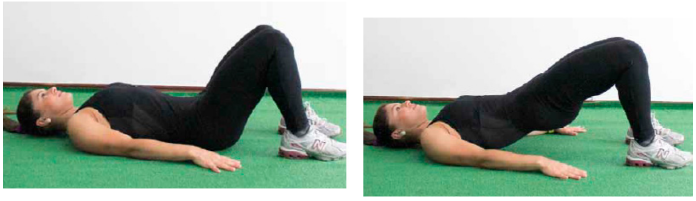

Exercise 3: *Sit to stand*

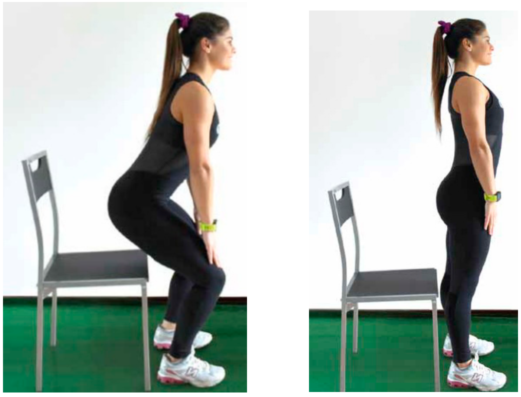

Exercise 4: *Shoulder Press*

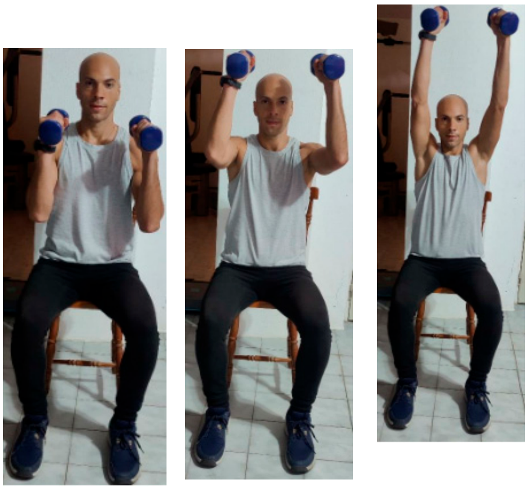

Exercise 5: *Biceps Curl*

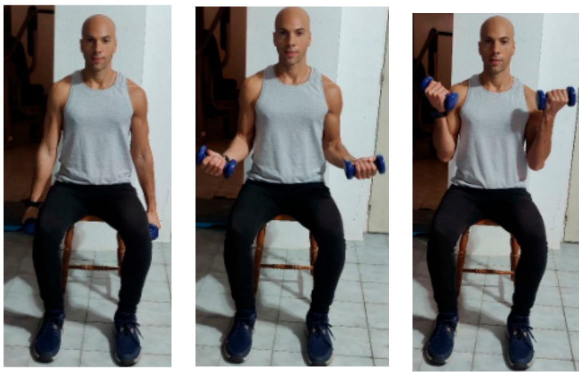

Exercise 6: *Calf Raises*

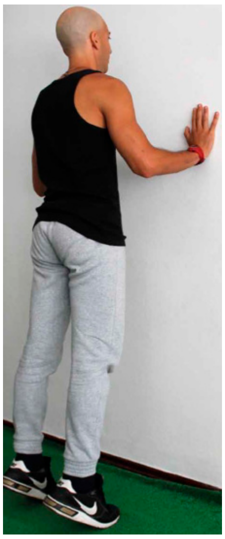

Table S3 Exercise adherence and adverse events (individual patient data)

**Patient no.**	**Length** (weeks)	**Type**	**Attendance rate**	**Total training volume**	**Compliance rate**	**Weekly training volume** (average)	**Average intensity** (RPE* and external load)	**Adverse events**
**1**	2 weeks	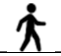	**217%**<br>(13 of 6 sessions)	**585 min**	**325%**	**195 min**	3	**No adverse events**
		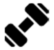	**125%**<br>(5 of 4 sessions)	**900 reps**	**125%**	**450 reps**	3.5<br>2 kg	
**2**	3 weeks	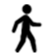	**100%**<br>(9 of 9 sessions)	**300 min**	**100%**	**100 min**	4.6	**No adverse events**
		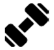	**100%**<br>(6 of 6 sessions)	**1260 reps**	**100%**	**420 reps**	5<br>2 kg	
**3**	3 weeks	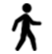	**89%**<br>(8 of 9 sessions)	**240 min**	**80%**	**80 min.**	3	**Grade 1:** Mild pain in extremity (foot) after walking; not limiting ADL **(**1/14 sessions)
		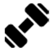	**100%**<br>(6 of 6 sessions)	**1080 reps**	**86%**	**360 reps**	3<br>1 kg	
**4**	3 weeks	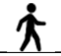	**100%**<br>(9 of 9 sessions)	**540 min**	**180%**	**180 min**	3.3	**No adverse events**
		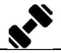	**150%**<br>(9 of 6 sessions)	**1620 reps**	**129%**	**540 reps**	3.3<br>2 kg	
**5**†	3 weeks	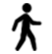	**89%**<br>(8 of 9 sessions)	**320 min**	**107%**	**107 min**	3.5	**No adverse events**
		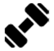	**100%**<br>(6 of 6 sessions)	**1260 reps**	**100%**	**420 reps**	3.3<br>2 kg	
**6**†	3 weeks	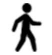	**100%**<br>(9 of 9 sessions)	**330 min**	**110%**	**110 min.**	3	**Grade 1:** Arthralgia (shoulder join) during shoulder press; mild pain, not limiting ADL **(**2/15 sessions)<br>Obs: Exercise was modified to lateral raises
		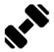	**100%**<br>(6 of 6 sessions)	**1260 reps**	**100%**	**420 reps**	3<br>2 kg	

Table S3 Exercise adherence and adverse events (individual patient data) (continued)

Patient no.	Length (weeks)	Type	Attendance rate	Total training volume	Compliance rate	Weekly training volume (average)	Average intensity (RPE* and external load)	Adverse events
**7**	4 weeks	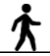	**100%**<br>(12 of 12 sessions)	**580 min**	**138%**	**145 min**	4	**No adverse events**
		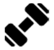	**100%**<br>(8 of 8 sessions)	**1800 reps**	**100%**	**450 reps**	3.5<br>2 kg	
**8**	6 weeks	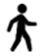	**111%**<br>(20 of 18 sessions)	**755 min**	**114%**	**126 min**	3.8	**No adverse events**
		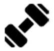	**150%**<br>(18 of 12 sessions)	**3330 reps**	**116%**	**555 reps**	3.7<br>1 kg	
**9**	3 weeks	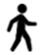	**78%**<br>(7 of 9 sessions)	**250 min**	**83%**	**83 min**	3.1	**No adverse events**
		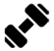	**117%**<br>(7 of 6 sessions)	**1440 reps**	**114%**	**480 reps**	3.3<br>1 kg	
**10***	4 weeks	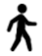	**83%**<br>(10 of 12 sessions)	**600 min**	**143%**	**150 min**	2.7	**Grade 1:** Leg muscle soreness after walking; mild symptoms, not limiting ADL (1/18 sessions)
		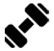	**100%**<br>(8 of 8 sessions)	**1935 reps**	**108%**	**484 reps**	2.9<br>2 kg	**Grade 2:** Arthralgia (knee join) after resistance exercise: Moderate pain; limiting instrumental ADL **(**1/18 sessions)
**11**	3 weeks	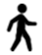	**100%**<br>(9 of 9 sessions)	**300 min**	**100%**	**100 min**	3.9	**Grade 1:** Leg muscle soreness after walking; mild symptoms, not limiting ADL **(**2/15 sessions)
		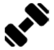	**100%**<br>(6 of 6 sessions)	**1500 reps**	**119%**	**500 reps**	3.5<br>2 kg	**Grade 1:** Arthralgia (shoulder join) during shoulder press; mild pain, not limiting ADL (1/15 sessions)

TableS3 Exercise adherence and adverse events (individual patient data) (continued)

**Patient no.**	**Length** (weeks)	**Type**	**Attendance rate**	**Total training volume**	**Compliance rate**	**Weekly training volume** (average)	**Average intensity** (RPE* and external load)	**Adverse events**
**12****	**2 weeks**		**100%** (5 of 5 sessions)	**80 min**	**53 %**	**40 min.**	1.5	**No adverse events**
			**100%** (4 of 4 sessions)	**810 reps**	**113%**	**405 reps**	2.3<br>1kg	
**13**	3 weeks		**100%** (9 of 9 sessions)	**300 min**	**100%**	**100 min.**	4.1	**Grade 1:** Arthralgia (shoulder join) during shoulder press; mild pain, not limiting ADL (1/15 sessions)
			**117%** (7 of 6 sessions)	**1530 reps**	**121%**	**510 reps**	4<br>1kg	
**14*****	3 weeks		**78%** (7 of 9 sessions)	**210 min.**	**70%**	**70 min.**	5	**No adverse events**
			**117%** (7 of 6 sessions)	**1260 reps**	**100%**	**420 reps**	3<br>2kg	

Legend: ADL (activities of daily living); obs. (observation); min. (minutes); kg (kilograms); reps (repetitions); RPE (rate of perceived exertion)

Aerobic exercise; Resistance exercise

Higher than the planned training volume Planned training volume Lower than the planned training volume

† Completed the exercise training program but were declared unsuitable for surgical resection intraoperatively (advanced-stage disease) and were not assessed after surgery.

*Required exercise modification due to preexisting knee osteoarthrosis (sit to stand was modified to standing hip flexion)

** Required exercise modification and dose reduction due to preexisting knee osteoarthrosis. Step up was modified to standing hip flexion and the prescribed aerobic training volume was lower than planned (initial dose of 30 minutes per week with an increment of 10 minutes per session during week 2).

*** Required the prescription of intermittent walking (three 10 minutes bouts per day) due to preexisting hip pain limiting walking long distances.

Table S4 Acceptability of the home-based exercise program

Acceptability components*	Question	**Median score** (mínimum-maximum) Scores 0-5 (5 = highest acceptability)
**Affective attitude**	What is your overall level of satisfaction with the exercise program?	**5** (4-5)
**Perceived benefit**	Do you consider that the exercise program has brought you any physical or mental benefit?	**5** (4-5)
	To what extent was the exercise program important in your preparation for surgical treatment?	**5** (4-5)
**Burden**	Was the weekly time required to perform the exercises adequate to the time that you had available?	**5** (4-5)
	Was the weekly time required to complete the exercise diary adequate to the time you had available?	**5** (5-5)
	Was the intensity of the exercise program adequate to your physical and mental capacity?	**5** (3,5)
**Intervention coherence**	Was the information initially given by the physical therapist adequate to understand the goals and content of the exercise program?	**5** (5-5)
	Was the information initially given by the physical therapist adequate to assess the rate of perceived exertion?	**5** (5-5)
	Was the weekly telephone supervision important to perform the exercise program?	**5** (1-5)
	Was the exercise guide important to perform the exercise program?	**5** (4-5)
**Self-efficacy**	How confidence did you feel in your ability to perform the walking training?	**5** (3-5)
	How confidence did you feel in your ability to perform the strength exercises?	**5** (5-5)
	How confident do you feel in continuing the exercise program after surgery?	**5** (4-5)

*Components were selected based on the theoretical framework proposed by Sekhon et al. (2017) [50]

References

29. Fletcher, G.F.; Ades, P.A.; Kligfield, P.; Arena, R.; Balady, G.J.; Bittner, V.A.; Coke, L.A.; Fleg, J.L.; Forman, D.E.; Gerber, T.C.; et al. Exercise standards for testing and training: a scientific statement from the American Heart Association. *Circulation* **2013**, *128*, 873-934, doi:10.1161/CIR.0b013e31829b5b44.
30. de Rooij, M.; van der Leeden, M.; Avezaat, E.; Häkkinen, A.; Klaver, R.; Maas, T.; Peter, W.F.; Roorda, L.D.; Lems, W.F.; Dekker, J. Development of comorbidity-adapted exercise protocols for patients with knee osteoarthritis. *Clinical interventions in aging* **2014**, *9*, 829-842, doi:10.2147/cia.s55705.
31. Riebe, D.; Franklin, B.A.; Thompson, P.D.; Garber, C.E.; Whitfield, G.P.; Magal, M.; Pescatello, L.S. Updating ACSM's Recommendations for Exercise Preparticipation Health Screening. *Med Sci Sports Exerc* **2015**, *47*, 2473-2479, doi:10.1249/mss.0000000000000664.
37. Campbell, K.L.; Winters-Stone, K.M.; Wiskemann, J.; May, A.M.; Schwartz, A.L.; Courneya, K.S.; Zucker, D.S.; Matthews, C.E.; Ligibel, J.A.; Gerber, L.H.; et al. Exercise Guidelines for Cancer Survivors: Consensus Statement from International Multidisciplinary Roundtable. *Medicine and science in sports and exercise* **2019**, *51*, 2375-2390, doi:10.1249/MSS.0000000000002116.
50. Sekhon, M.; Cartwright, M.; Francis, J.J. Acceptability of healthcare interventions: An overview of reviews and development of a theoretical framework. *BMC Health Services Research* **2017**, *17*, doi:10.1186/s12913-017-2031-8.
